# The taxonomic status of *Myotisnesopoluslarensis* (Chiroptera, Vespertilionidae) and new insights on the diversity of Caribbean *Myotis*

**DOI:** 10.3897/zookeys.1015.59248

**Published:** 2021-02-10

**Authors:** Roberto Leonan M. Novaes, Vinícius C. Cláudio, Roxanne J. Larsen, Don E. Wilson, Marcelo Weksler, Ricardo Moratelli

**Affiliations:** 1 Universidade Federal do Rio de Janeiro, Programa de Pós-Graduação em Biodiversidade e Biologia Evolutiva. Av. Carlos Chagas Filho 373, Cidade Universitária, 21941-902, Rio de Janeiro, RJ, Brazil; 2 Smithsonian Institution, National Museum of Natural History, Division of Mammals. 10th St. & Constitution Ave. NW, 20013-7012, Washington, DC, USA; 3 University of Minnesota, College of Veterinary Medicine, 1365 Gortner Ave., 55108, Saint Paul, MN, USA; 4 Museu Nacional / Universidade Federal do Rio de Janeiro, Departamento de Vertebrados. Quinta da Boa Vista s/n, São Cristóvão, 20940-040, Rio de Janeiro, RJ, Brazil; 5 Fundação Oswaldo Cruz, Fiocruz Mata Atlântica. R. Sampaio Correa s/n, Taquara, 22713-560, Rio de Janeiro, RJ, Brazil

**Keywords:** Bats, biogeography, Lesser Antilles, morphology, morphometry, taxonomy, South America, Venezuela

## Abstract

*Myotisnesopolus* currently comprises two subspecies. The nominate subspecies (*M.n.nesopolus*) occurs on the Caribbean islands of Curaçao and Bonaire, Netherlands Antilles, whereas *M.n.larensis* is known from mainland South America in northeastern Colombia and northwestern Venezuela. Our Maximum Likelihood phylogenetic analyses of cytochrome-b gene sequences recovered *M.nesopolus* as a paraphyletic group, with *M.n.nesopolus* and *M.n.larensis* as non-sister lineages. The haplotype network indicates that these two subspecies do not share any haplotypes and are in different evolutionary trajectories. Additionally, these two subspecies can be distinguished on the basis of qualitative and quantitative morphological traits. This pattern supports the recognition of *M.nesopolus* and *M.larensis* as full species. Our results also reveal that the assemblage of Caribbean *Myotis* do not form a monophyletic group. Caribbean species are phylogenetically close to mainland species from northern South America and Central America, suggesting that colonization of Caribbean islands happened multiple times.

## Abstract (pt)

Atualmente *Myotisnesopolus* compreende duas subespécies: *M.n.nesopolus* ocorre nas ilhas caribenhas de Curaçao e Bonaire, Antilhas Holandesas, enquanto *M.n.larensis* é conhecido para o continente da América do Sul, no nordeste da Colômbia e noroeste da Venezuela. Nossa inferência filogenética por Máxima Verossimilhança recuperou *M.nesopolus* como parafilética, com *M.n.nesopolus* e *M.n.larensis* sendo linhagens não-irmãs. Além disso, essas duas subespécies não compartilham nenhum haplótipo. Adicionalmente, as subespécies podem ser diferenciadas a partir de caracteres morfológicos e morfométricos. Esse achado suporta o reconhecimento de *M.nesopolus* e *M.larensis* como espécies distintas. Nossos resultados revelam que os *Myotis* do Caribe não formam um grupo monofilético. Espécies caribenhas são filogeneticamente próximas de espécies continentais das Américas Central e do Sul, sugerindo que a colonização das ilhas do Caribe aconteceu por múltiplos eventos de dispersão.

## Introduction

*Myotis* Kaup, 1829 (Vespertilionidae, Myotinae) comprises more than 120 species distributed worldwide, and is the most speciose genus of bats (Simmons 2005; Burgin et al. 2018). Twenty-seven species are recognized from the Neotropics (Wilson 2008; Moratelli et al. 2017, 2019a; Carrión-Bonilla and Cook 2020). However, molecular evidence has revealed that the current species richness is underestimated (Clare et al. 2011; Larsen et al. 2012a; Chaverri et al. 2016; Moratelli et al. 2017).

Two subspecies of *Myotisnesopolus* Miller, 1900 are recognized. The nominate subspecies, *M.n.nesopolus*, is known from Curaçao and Bonaire in the Netherlands Antilles. The other subspecies, *M.n.larensis* LaVal, 1973, is known from mainland South America in northeastern Colombia and northwestern Venezuela (LaVal 1973; Wilson 2008; Muñoz-Garay and Mantilla-Meluk 2012; Moratelli et al. 2013). LaVal (1973) described *Myotislarensis* as a full species from “Río Tocuyo, Lara, Venezuela”. Genoways and Williams (1979), however, treat *larensis* as a subspecies of *Myotisnesopolus*. Miller’s (1900) description of *M.nesopolus* was based on one specimen from Willemstad, Curaçao, Netherlands Antilles. Subsequently, Genoways and Williams (1979) considered that representatives of *Myotis* from Bonaire island, originally identified as *Myotisnigricans* (Schinz, 1821), were misidentifications of *M.nesopolus*, which was confirmed by Moratelli et al. (2017).

Previous molecular and morphological studies questioned the subspecific status of mainland populations of *M.nesopolus*, suggesting that the two subspecies might represent different species (Larsen et al. 2012b; Moratelli et al. 2013, 2017). Here we reassess the taxonomic status of *M.n.larensis* in the light of new morphological and genetic analyses.

## Materials and methods

### Specimens examined

Specimens of *M.nesopolus* used in this study are deposited in the American Museum of Natural History (**AMNH**, New York, USA), Carnegie Museum of Natural History (**CM**, Pittsburgh, USA), Smithsonian’s National Museum of Natural History (**USNM**, Washington DC, USA), and Museum of Texas Tech University (**TTU**, Lubbock, USA). We examined the holotype of *M.n.nesopolus* (USNM 101849), two topotypes from Curaçao (CM 52432, USNM 105128), and nine specimens from Bonaire (Appendix 1). Material of *M.n.larensis* includes the holotype (AMNH 130709), and fifteen additional specimens from mainland Venezuela.

### Molecular analyses

Phylogenetic analyses of complete cytochrome-b gene (cyt-b, 1,140 bp, no gaps) sequences were conducted for the Neotropical assemblage of *Myotis*. A total of 122 sequences, including outgroups, were retrieved from GenBank (Appendix 2). We used the palearctic species *Myotisbrandtii* (Eversmann, 1845) and *Myotisgracilis* Ognev, 1927 as outgroups because they are sister to the Neotropical clade (see Ruedi et al. 2013). Multiple sequence alignment of full length cyt-b sequences were performed with MEGA X (Kumar et al. 2018), using MUSCLE algorithm with default settings (Edgar 2004). Subsequently, the Bayesian Information Criterion (BIC), as implemented in JModelTest2 (Darriba et al. 2012), was used to determine the best-fit models of nucleotide substitution. The Hasegawa-Kishino-Yano model (Hasegawa et al. 1985) was chosen to correct the heterogeneity rate using gamma-distribution with invariant sites (i.e., HKY + Γ + I).

The phylogenetic analysis was carried out using Maximum Likelihood (ML) method (Felsenstein 1981), in the software RAxML v8.0 (Stamatakis 2014). To assess the nodal support, we calculated a nonparametric bootstrap using 1000 replications. Genetic distance values for cyt-b sequences were calculated in MEGA X using the Kimura 2-parameter model (Kimura 1980).

To understand the population structure of *M.n.nesopolus*, *M.n.larensis* and other phylogenetically related population groups, we built a haplotype network (distribution of haplotypes by previously defined population groups) using the median-joining algorithm in the Network 4.6.1.3 software (Bandelt et al. 1999).

### Morphological and morphometric analyses

We examined 284 specimens for the morphological comparisons, including *M.n.nesopolus* (*N* = 10), *M.n.larensis* (*N* = 9) and 14 species of Neotropical *Myotis* deposited in 11 collections in Brazil, Canada and United States (Appendix 1). Specimens were identified following Wilson (2008) and Moratelli et al. (2011, 2013, 2017). The main qualitative morphological characters used in the comparisons were: (i) presence and height of sagittal crest; (ii) presence and height of lambdoidal crests; (iii) inclination shape of the frontal and parietal bones; (iv) presence of a fringe of hairs along the trailing edge of the uropatagium; (v) dorsal and ventral fur texture and height; (vi) pattern of fur coloring, with the capitalized color nomenclature following Ridgway (1912).

We took one external and 16 craniodental measurements (Table 1), using digital calipers to the nearest 0.01 mm. Measurements were made under binocular microscopes with low magnification (usually 6×). Measurements were recorded from adults and are reported in millimeters (mm). The length of ear and body mass were recorded from skin labels. We used a principal component analysis (PCA) to identify general trends of cranial size and shape variation among samples, and a discriminant function analysis (DFA), with a priori identification of samples, to compare skull size and shape of *M.n.nesopolus* (*N* = 9) and *M.n.larensis* (*N* = 9). For these analyses, we selected a subset of 11 craniodental dimensions representing different axes of the length and width of skull, rostrum, and mandible, as follows: greatest length of skull, including incisors (GLS), condylo-incisive length (CIL), mastoid breadth (MAB), braincase breadth (BCB), interorbital breadth (IOB), postorbital breadth (POB), breadth across canines (BAC), breadth across molars (BAM), maxillary toothrow length (MTL), molariform toothrow length (M1–M3), and mandibular toothrow length (MAN). PCA and DFA analyses were run in R software (R Development Core Team 2012) using the MASS and Lattice packages (Venables and Ripley 2002; Sarkar 2008). Because multivariate procedures require complete data sets, missing values (ca 1.5% of the total dataset) were estimated from the existing raw data using the Amelia II package (Honaker et al. 2011) implemented in R software. Measurements were transformed to natural logs and covariance matrices were computed considering all variables. Subsequently, an analysis of variance using Mann-Whitney statistics was employed to test whether the population samples differ in cranial dimensions. The comparison was made using *p*-values and when less than 0.001 were considered as statistically significant. This analysis was run in the software PAST 3.3 (Hammer et al. 2001).

**Table 1.** Description of cranial, mandibular, and external dimensions (and their abbreviations). Lengths were measured from the anteriormost point or surface of the 1^st^ structure to the posteriormost point or surface of the 2^nd^ structure, except as specified.

Measurements	Acronyms	Descriptions
Forearm length	FA	From the elbow to the distal end of the forearm including carpals
Greatest length of skull	GLS	From the apex of the upper internal incisors, to the occiput
Condylo-canine length	CCL	From the anterior surface of the upper canines to a line connecting the occipital condyles
Condylo-basal length	CBL	From the premaxillae to a line connecting the occipital condyles
Condylo-incisive length	CIL	From the apex of upper internal incisors to a line connecting the occipital condyles
Basal length	BAL	Least distance from the apex of upper internal incisors to the ventral margin of the foramen magnum
Zygomatic breadth	ZYG	Greatest breadth across the outer margins of the zygomatic arches
Mastoid breadth	MAB	Greatest breadth across the mastoid region
Braincase breadth	BCB	Greatest breadth of the globular part of the braincase
Interorbital breadth	IOB	Least breadth between the orbits
Postorbital breadth	POB	Least breadth across frontals posterior to the postorbital bulges
Breadth across canines	BAC	Greatest breadth across outer edges of the crowns of upper canines, including cingulae
Breadth across molars	BAM	Greatest breadth across outer edges of the crowns of upper molars
Maxillary toothrow length	MTL	From the upper canine to M3
Molariform toothrow length	M1–M3	From M1 to M3
Mandibular length	MAL	From the mandibular symphysis to the condyloid process
Mandibular toothrow length	MAN	From the lower canine to m3

## Results

### Molecular analyses

**Figure 1.**
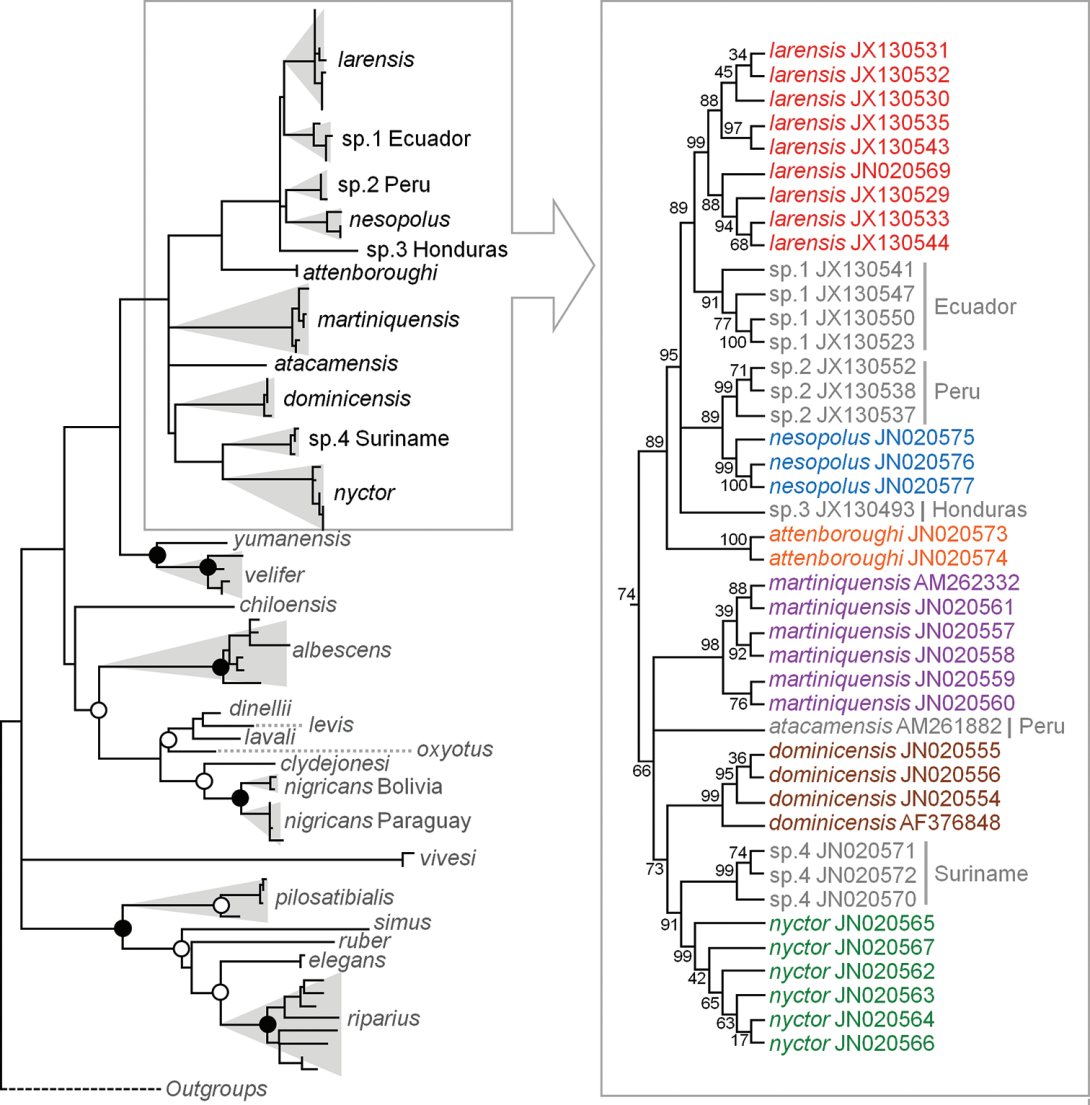
Phylogenetic tree resulting from the Maximum Likelihood analysis of cytochrome-b sequences of species of *Myotis*. Nodal support was calculated by bootstrap and black solid circles are values between 100–95% and hollow white circle are values between 94–90%. Values less than 90% were not indicated. The rectangle encloses the phylogenetic relationship, where branches were transformed to cladogram, among *M.nesopolus*, *M.larensis*, Caribbean *Myotis* (colored terminals) and mainland haplogroups of five more closely related species and candidate species.

The ML phylogeny based on cyt-b sequences indicates that *M.nesopolus*, as currently recognized, is paraphyletic, with *M.n.nesopolus* more closely related to an eastern Peruvian unidentified lineage, whereas *M.n.larensis* was recovered more closely related to an unidentified lineage from western Ecuador (Fig. 1), although this phylogeny and branching events has low nodal support. These unidentified species from Peru and Ecuador were originally designated as *Myotisnigricans* by the original collector due to morphological similarities. However, *M.nigricans* has been recovered as polyphyletic and considered a cryptic species complex in many studies (Moratelli et al. 2011, 2013, 2016, 2017; Larsen et al. 2012a). Therefore, we decided not to give a name to the lineages related to *M.nesopolus* and *M.larensis*. We emphasize that the previous identification of these specimens as *M.nigricans* by one of our authors (RJL) in a previous study (Larsen et al. 2012a) indicates that these populations are morphologically distinct from those considered here as *M.nesopolus* and *M.larensis*.

The Caribbean *Myotis* species do not form a monophyletic group, being related to *Myotisatacamensis* (Lataste, 1892) and other mainland putative species. Nevertheless, the phylogenetic relationship of Caribbean *Myotis* clade is not fully resolved, since a polytomy was recovered among *M.* sp. 3 from Honduras and the ancestral lineage of *M.n.nesopolus* and *M.* sp. 2 from Peru, and of *M.n.larensis* and *M.* sp. 1 from Ecuador. Similarly, a polytomy was recovered among *M.atacamensis*, *M.martiniquensis* and an ancestral lineage of *M.dominicensis*, *M.nyctor* and *M.* sp. 4 from Suriname (Fig. 1).

The average cyt-b pairwise distance between *M.n.larensis* and *Myotis* sp. 1 from western Ecuador is 2.1% ± 0.3; between *M.n.nesopolus* and *Myotis* sp. 2 from eastern Peru is 3.8% ± 0.4; and between *M.n.nesopolus* and *M.n.larensis* is 4.0% ± 0.3 (Table 2). Levels of intraspecific variation were less than 0.8% for all recognized and putative species (Table 2).

**Table 2.** Average Kimura 2-parameter genetic distances within (along diagonal) and among (below diagonal) *Myotis* taxa based on cytochrome-b gene sequences. Boldface value indicates the distance between *M.larensis* and *M.nesopolus*. Hyphen indicates groups with a single sequence.

Taxa		1	2	3	4	5	6	7	8	9	10	11	12
1	*M.atacamensis* (Peru)	–											
2	*Myotis* sp. 4 (Suriname)	0.085	0.002										
3	*M.nyctor* (Grenada)	0.103	0.080	–									
4	*M.nyctor* (Barbados)	0.089	0.070	0.002	0.004								
5	*M.dominicensis* (Dominica)	0.080	0.087	0.092	0.088	0.001							
6	*M.martiniquensis* (Martinique)	0.087	0.093	0.089	0.094	0.887	0.002						
7	*M.n.larensis* (Venezuela)	0.093	0.107	0.127	0.119	0.097	0.096	0.003					
8	*Myotis* sp. 1 (W Ecuador)	0.091	0.104	0.134	0.120	0.092	0.093	0.021	0.002				
9	*Myotis* sp. 2 (E Peru)	0.104	0.115	0.138	0.126	0.107	0.104	0.034	0.033	0.001			
10	*M.n.nesopolus* (Bonaire)	0.103	0.115	0.147	0.124	0.104	0.106	**0.040**	0.044	0.038	0.008		
11	*Myotis* sp. 3 (Honduras)	0.103	0.116	0.133	0.120	0.107	0.105	0.046	0.049	0.056	0.053	–	
12	*M.attenboroughi* (Tobago)	0.081	0.093	0.101	0.099	0.091	0.088	0.068	0.075	0.076	0.078	0.079	0.000

**Figure 2.**
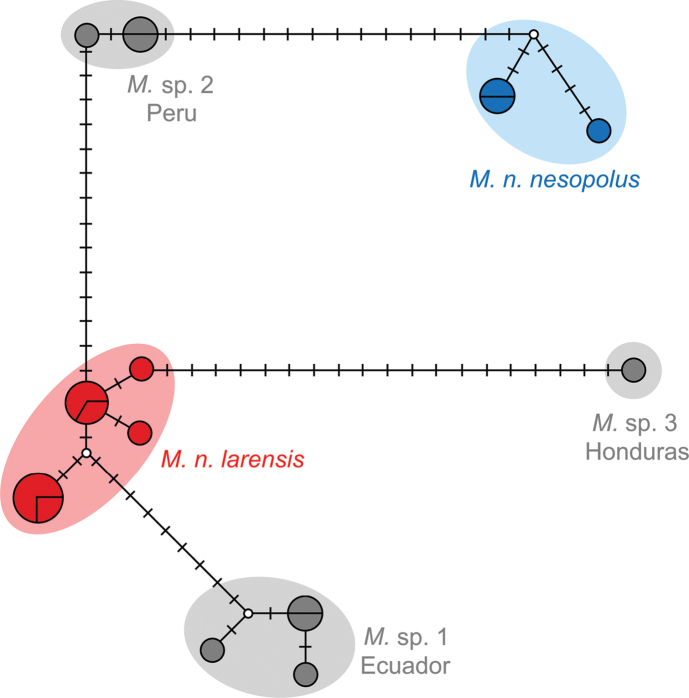
Haplotype network from cyt-b sequences of *Myotisnesopolus* (blue), *Myotislarensis* (red) and other mainland closest *Myotis* lineages from Central and South America. Each tick mark represents a single base-pair mutation.

The haplotype network indicates that there are no haplotypes shared between *M.n.nesopolus*, *M.n.larensis*, and phylogenetically close species (Fig. 2). The haplotypes were grouped into small clusters well-distributed among the populations, with no central haplotype. The network indicates spatial structuring with isolation among the population groups tested, agreeing with what was obtained by phylogenetic inference.

### Morphological analyses

**Figure 3.**
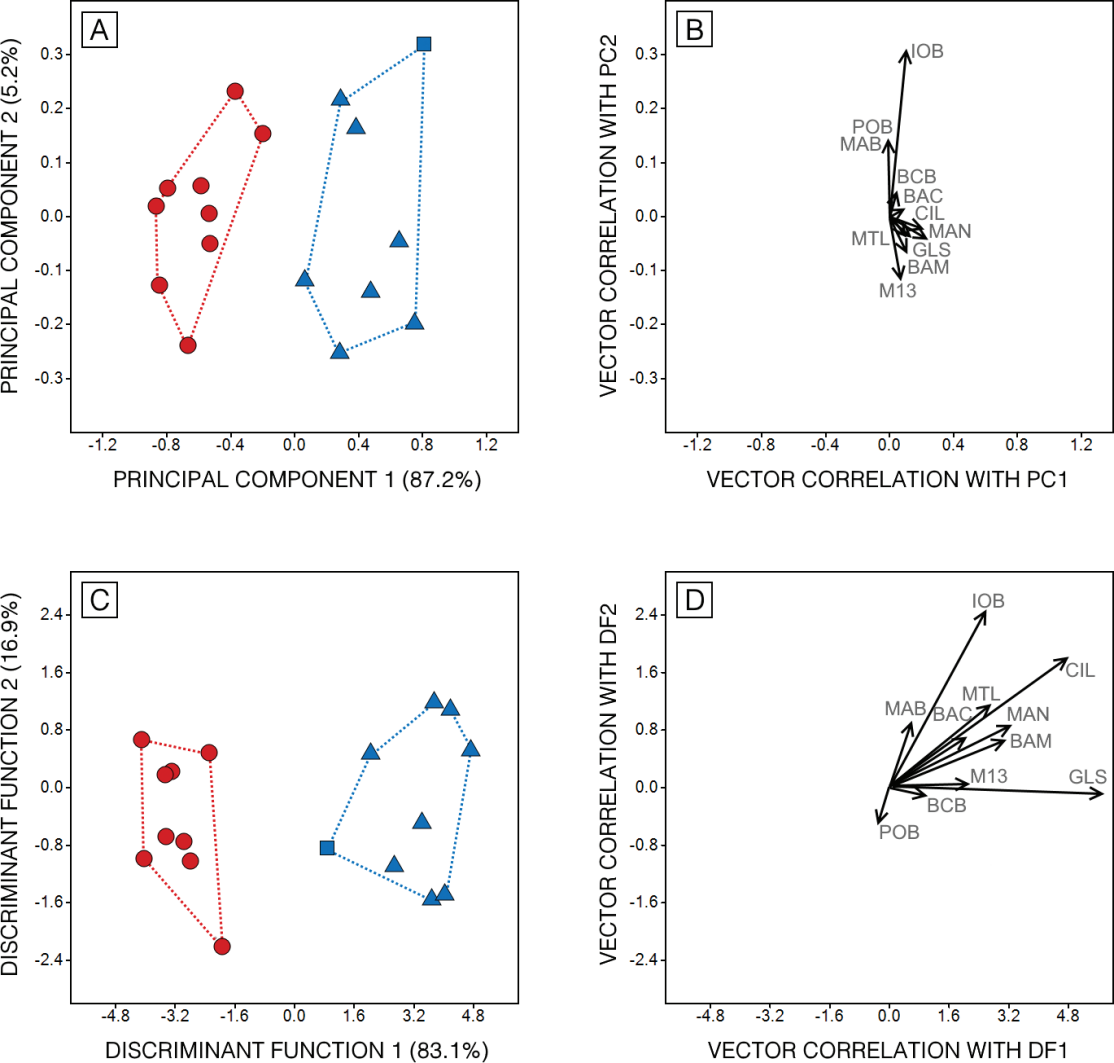
Plots showing convex-hulls and vector correlation of cranial measurements of Principal Component Analysis (**A, B**) and Discriminant Function Analysis (**C, D**) for *Myotisnesopolus* from Curaçao (black square), *Myotisnesopolus* from Bonaire (blue triangles) and *Myotislarensis* from Venezuela mainland (red dots).

The first principal component (PC1) accounted for 87% of the total craniometric variation, and represents overall skull size (Fig. 3A, B). Along this axis, scores of *M.n.larensis* and *M.n.nesopolus* do not overlap. On the other hand, the two samples overlap broadly along the second principal component (PC2 = 5%) which represents overall skull shape. The distribution of *M.n.larensis* and *M.n.nesopolus* samples across size and shape axes in the discriminant analysis (Fig. 3C, D) is similar to that observed in the PCA. Measurements associated with skull and mandible length (GLS, CIL, MAN) and skull width (IOB) were the most useful to discriminate samples (Table 3). Considering that skull axes are represented by the set of measurements used in the morphometric multivariate analysis, these results reveal that *M.n.larensis* and *M.n.nesopolus* have distinct skull size and shape.

**Table 3.** Vector correlation loadings with original variables of principal components (PC1 and PC2) and discriminant functions (DF1 and DF2) for selected samples of *M.larensis* and *M.nesopolus*. See Table 1 for variable abbreviations.

Measurements	PC 1	PC2	DF1	DF2
MAN	0.324	-0.091	0.063	0.016
GLS	0.573	-0.103	0.109	0.026
CIL	0.506	-0.056	0.093	0.027
MAB	0.097	0.327	0.012	0.012
BCB	0.109	0.108	0.019	0.003
IOB	0.258	0.775	0.051	0.014
POB	-0.02	0.363	-0.005	0.026
BAC	0.198	0.031	0.04	0.021
BAM	0.277	-0.165	0.059	-0.015
MTL	0.262	-0.088	0.052	0.011
M1–3	0.187	-0.298	0.040	-0.007

Populations from the Antilles and mainland South America do not overlap in measurements of several characters, which may be useful in distinguishing species: *M.n.larensis* forearm length ranges from 31.2 to 33.2 mm, and GLS from 13.6 to 14.5 mm; *M.n.nesopolus* forearm length ranges from 28.2 to 31.0 mm, and GLS from 12.9 to 13.4 mm. The Mann-Whitney test found significant differences in 11 of the 14 measurements tested (Table 4).

**Table 4.** Selected measurements (mm) of *M.larensis* from Venezuela and *M.nesopolus* from Curaçao and Bonaire. Descriptive statistics include the mean, range (in parentheses), and sample size. See Table 1 for variable abbreviations. Mann-Whitney Test *p*-values was used to compare cranial measurements between samples. Measurements with hyphen (–) not were tested due to disparate samples size.

Measurements	*Myotislarensis*	*Myotisnesopolus*	*P*–value
FA	32.2 (31.2–33.2) 7	29.7 (28.2–31.0) 11	–
GLS	13.7 (13.3–14.4) 9	12.9 (12.8–13.1) 9	< 0.001
CCL	12.1 (11.5–12.7) 9	11.6 (11.4–11.8) 9	< 0.001
CBL	12.8 (12.4–13.5) 9	12.2 (12.0–12.5) 9	< 0.001
CIL	12.9 (12.6–13.6) 9	12.4 (12.2–12.6) 9	< 0.001
BAL	11.6 (11.2–12.4) 9	11.1 (10.9–11.3) 9	< 0.001
ZYG	8.1 (8.0–8.2) 3	7.8 (7.7–8.0) 8	–
MAB	5.3 (5.1–5.6) 9	6.7 (6.4–6.8) 9	0.247
BCB	6.2 (6.1–6.3) 9	6.1 (5.9–6.2) 9	0.017
IOB	4.4 (4.0–4.7) 9	4.0 (3.9–4.2) 9	0.003
POB	3.3 (3.2–3.4) 9	3.3 (3.2–3.5) 9	0.374
BAC	3.3 (3.2–3.5) 9	3.0 (3.0–3.2) 9	< 0.001
BAM	5.3 (5.1–5.5) 9	4.9 (4.8–5.0) 9	< 0.001
MTL	5.2 (5.0–5.4) 9	4.8 (4.7–4.9) 9	< 0.001
M1M3	2.9 (2.8–3.2) 9	2.7 (2.6–2.8) 9	< 0.001
MAL	9.8 (9.5–10.3) 4	9.0 (8.8–9.2) 9	–
MAN	5.5 (5.3–5.9) 8	5.1 (4.9–5.3) 9	< 0.001

**Figure 4.**
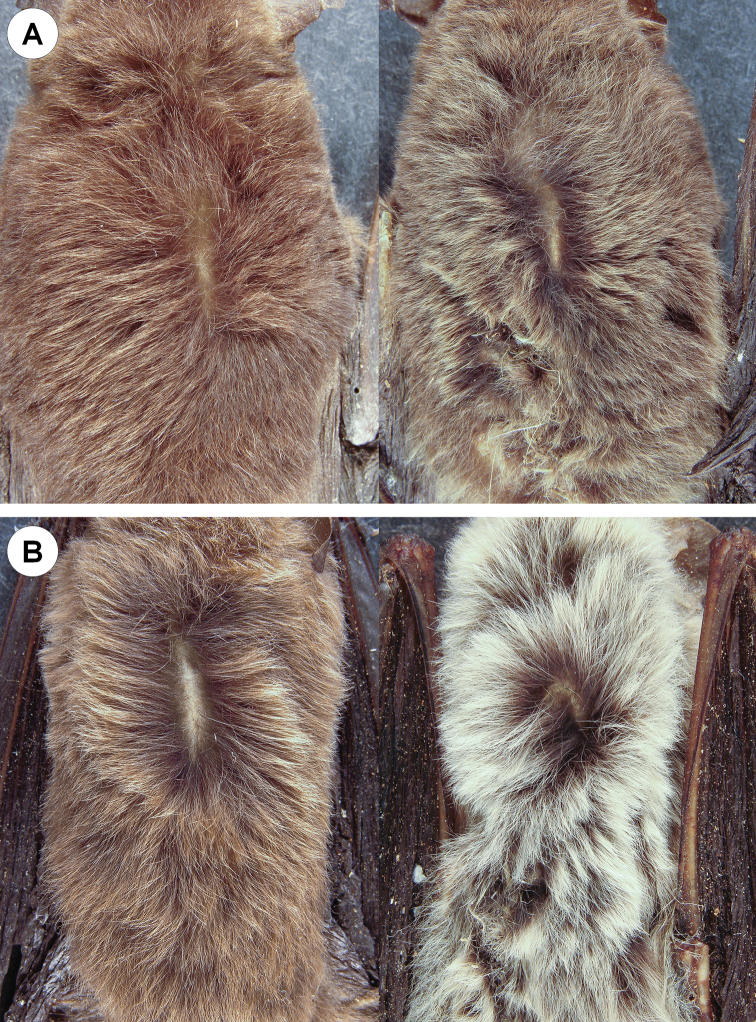
Dorsal (left) and ventral (right) fur of a specimen of *Myotisnesopolus* (CM 52217 [**A**]) from Bonaire and the holotype of *Myotislarensis* (USNM 441737 [**B**]) from Lara, Venezuela.

**Figure 5.**
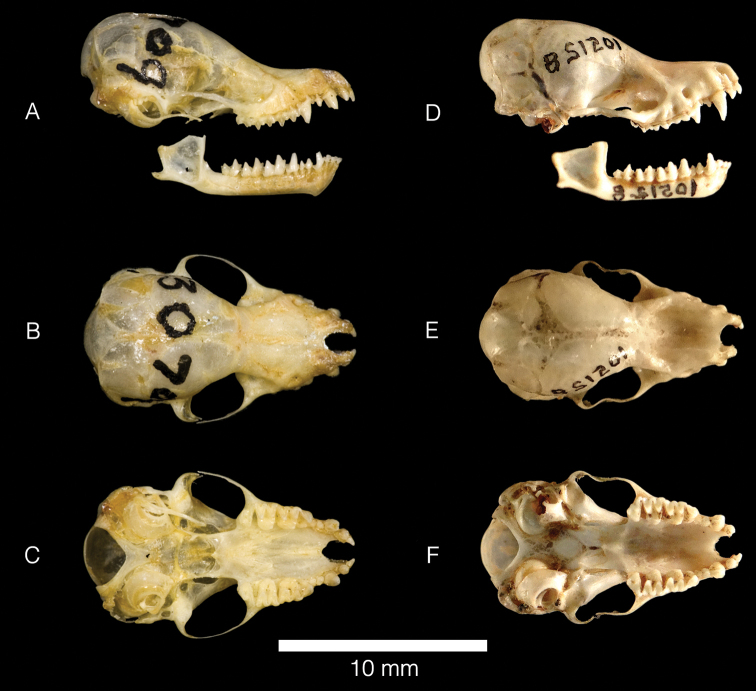
Skull profiles of *Myotislarensis* (AMNH 130709 [holotype]) from Venezuela in lateral (**A**), ventral (**B**) and dorsal (**C**) views; and *Myotisnesopolus* (USNM 105128 [topotype]) from Curaçao in lateral (**D**), ventral (**E**) and dorsal (**C**) views. The image of the *M.nesopolus* skull was inverted.

Population samples from the Antilles and mainland South America have several qualitative morphological differences. Specimens of *M.n.nesopolus* have moderately silky fur (length of dorsal fur 5–6 mm; length of ventral fur 3–4 mm); dorsal fur Dresden-Brown with little contrast between bases and tips slightly lighter tips; ventral fur with blackish bases and Light-Buff tips (Fig. 4A). Specimens of *M.n.larensis* have long silky fur (length of dorsal fur 6–8 mm; length of ventral fur 5–6 mm); dorsal fur strongly bicolored, with blackish bases (2/3) and Tawny-Olive tips (1/3); ventral fur with blackish bases and whitish tips (Fig. 4B). The sagittal crest is absent in *M.n.nesopolus*, the lambdoidal crests are generally absent or very low, and the parietal is inclined forward. Sagittal and lambdoidal crests are present in *M.n.larensis*, ranging from low to moderate in development, and the parietal is not inclined forward. In both populations, the second upper premolar (P3) is aligned in the toothrow and visible in labial view, and the occipital region is always rounded (Fig. 5).

The congruence between the molecular and morphological evidence indicates that the two subspecies of *M.nesopolus* do not form a clade. Thus, *M.larensis* represents an independent evolutionary lineage and should be treated as a full species.

### Description and comparisons

*Myotislarensis* is a small-sized bat (total length 78–82 mm; forearm length 31.2–33.2; body mass 3–5 g), morphologically similar to several Neotropical congeners. Ears are moderate in size (length 10–13 mm), and when laid forward extend halfway from eye to nostril. Antitragal notch is barely evident. Membranes are Mummy-brown. Fur on dorsal surface of uropatagium extends slightly past the knees. Plagiopatagium is attached to the foot at toes level by a broad band of membrane. Third metacarpal, tibia, and skull are long in relation to forearm (mean ratios 0.96, 0.48, and 0.43, respectively; see LaVal (1973)).

*Myotislarensis* can be distinguished from all Caribbean and South American congeners by qualitative and quantitative traits. It differs from *M.nesopolus* by its larger size (no overlapping in forearm length and greatest length of skull), presence of sagittal crest, and dorsal fur longer and strongly bicolored. Considering the *Myotis* species that occurs in the northern South America, *M.larensis* differs from *M.albescens* (É. Geoffroy, 1806) by the absence of a fringe of hairs along the trailing edge of the uropatagium; from *M.keaysi* J. A. Allen, 1914, *M.pilosatibialis* LaVal, 1973, *M.riparius* Handley, 1960, and *M.simus* Thomas, 1901 by the long silky dorsal fur strongly bicolored. *Myotislarensis* can also be distinguished from *M.simus* by the plagiopatagium broadly attached at base of the toes. *Myotislarensis* differs from *M.diminutus* Moratelli & Wilson, 2011 by its larger cranial dimensions and dorsal fur strongly contrasting; from *M.handleyi*Moratelli et al., 2013 by its strongly contrasting and long silk dorsal fur and shorter forearm; from *M.oxyotus* (Peters, 1867) by having a smaller skull, less steeply sloping frontals and strongly contrasting dorsal fur. *Myotislarensis* differs from *M.attenboroughi*Moratelli et al., 2017 by its lighter and strongly contrasting dorsal fur and larger skull; and from *M.clydejonesi*Moratelli et al., 2016 by its moderate steeply sloping frontals, less inflated braincase, smaller skull and dorsal fur strongly contrasting. *Myotislarensis* differs from *M.caucensis* Allen, 1914 by its smaller skull and strongly contrasting dorsal fur. *Myotislarensis* can be distinguished from M.cf.nigricans from northern South America (sensu Moratelli et al. 2013) by the lighter dorsal and ventral fur, more developed sagittal and lambdoid crests and parietal not inclined forward.

## Discussion

Genoways and Williams (1979) determined that mainland and island specimens of *M.larensis* and *M.nesopolus*, respectively, were morphometrically similar, with Venezuelan specimens slightly smaller than those from Curaçao. As a result, they recognized *M.larensis* as a subspecies of *M.nesopolus*, which was followed by subsequent authors (e.g., Simmons 2005; Wilson 2008; Moratelli et al. 2019b). However, our results do not support this arrangement, indicating a morphometric discontinuity and qualitative morphological differences between *M.larensis* and *M.nesopolus*.

Previous phylogenetic studies based on mitochondrial and nuclear DNA recovered *M.nesopolus* and *M.larensis* as sister lineages and questioned the subspecific status of *M.larensis* because the cyt-b genetic distance of 4% between mainland and Antilles populations suggests a potential for separation at the species level (see Bradley and Baker 2001; Larsen et al. 2012b). However, this study did not include the mainland samples from Ecuador and Peru. Our phylogenetic analyses revealed that *M.nesopolus* and *M.larensis* are not sister lineages and do not share haplotypes. The genetic distances between *M.nesopolus*, *M.larensis* and their sister species are greater than 2%. About this, Bradley and Baker (2001) indicate that genetic distance values between 2 and 11% from cyt-b sequences had a high probability of being indicative of conspecific populations or valid species and merit additional study concerning specific status. Our investigation found a conspicuous phenotypic discontinuity in variation of both the size and shape of the skull and other external characters. Thus, the strong congruence between the morphological, morphometric and molecular evidence presented here supports the hypothesis that *M.larensis* represents a full species.

Nevertheless, it is important to mention the limitation of cyt-b gene for establishing species boundaries in the Caribbean clade, particularly between *M.larensis* and *M.* sp. 1 from Ecuador and between *M.nesopolus* and *M.* sp. 2 from Peru. Although widely used (e.g., Larsen et al. 2012a, b; Moratelli et al. 2016, 2017; Carrión-Bonilla and Cook 2020), the application of cyt-b data to species delimitation and inference of phylogenetic relationships in *Myotis* from the Caribbean clade was insufficient. This demonstrates the need to expand the use of new genetic markers for future systematic studies with the Caribbean *Myotis* assemblage.

**Figure 6.**
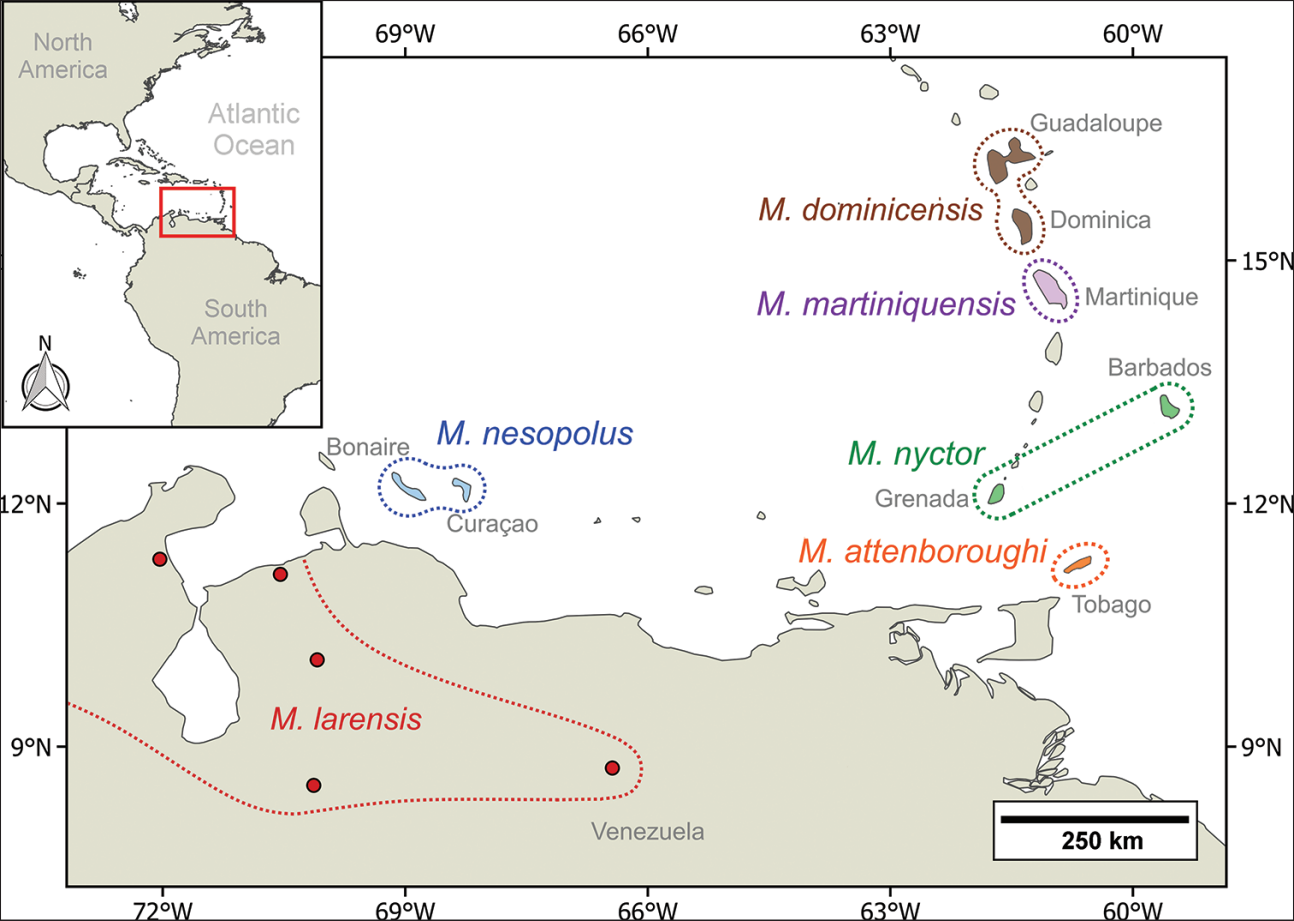
Geographic distributions of *Myotislarensis* (restricted to mainland South America in Venezuela and Colombia) and Caribbean *Myotis* species *M.nesopolus*, *M.dominicensis*, *M.martiniquensis*, *M.nyctor*, and *M.attenboroughi*.

With the recognition of *M.larensis* at the species level hierarchy, *M.nesopolus* is restricted to Bonaire and Curaçao and is the only species of the genus found in these islands (Fig. 6). Similarly, other Caribbean islands have unique *Myotis* species, including: *Myotisdominicensis* Miller, 1902 restricted to Dominica and Guadeloupe; *Myotismartiniquensis* LaVal, 1973 is restricted to Martinique; *Myotisattenboroughi* is restricted to Tobago; and *Myotisnyctor* LaVal & Schwartz, 1974 is restricted to Barbados and Grenada (LaVal 1973; Larsen et al. 2012a; Moratelli et al. 2017). However, the taxonomic status of some populations of these species needs to be reassessed. For example, *Myotisnyctor* was described from Barbados and subsequently recorded from Grenada (LaVal 1973; LaVal and Schwartz 1974; Moratelli et al. 2017). Although our phylogenetic analysis grouped the samples of *M.nyctor* from Barbados (*N* = 5) and Grenada (*N* = 1) in the same clade (Fig. 1), and with low genetic distance between them (ca 0.2%; Table 2), there are qualitative and quantitative morphological differences between specimens from these two islands (see Larsen et al. 2012a). The similarity in the cyt-b sequences between Grenada and Barbados specimens may be explained by the retained ancestral polymorphism due to the very recent separation (Stadelmann et al. 2007; Larsen et al. 2012a).

The biogeographic interpretations made by Larsen et al. (2012b) suggest at least two independent *Myotis* invasions into the Lesser Antilles, and reverse colonization by Caribbean *Myotis* to mainland Central and South America—the latter being a well-documented pattern in other Caribbean bat lineages (Dávalos 2005, 2006, 2010; Genoways et al. 2005; Pavan and Marroig 2017; Tavares et al. 2018). In addition, some biogeographic and ecological aspects suggest the need for taxonomic revision of some species. The distance and geographic isolation between Barbados and Grenada (ca 255 km) are greater than between Dominica and Martinique (ca 42 km), each one having a unique *Myotis* species. Moreover, Barbados and Grenada are separated by the Tobago Basin, with an ocean depth of approximately 2500 m and no ridges that may have connected these two populations during glaciation periods (Speed 1981; Humphrey 1997; Graham 2003). Considering the apparent low vagility and the small home range of *Myotis* in general (e.g., LaVal and Fitch 1977; Castella et al. 2001; Moratelli et al. 2019b), it is possible that the populations of *M.nyctor* from these two islands are isolated and on different evolutionary trajectories. The same rationale might be valid for *M.dominicensis*, where the populations from Guadeloupe and Martinique are isolated by approximately 42 km of sea. However, there are several oceanic ridges between these two islands, which may have served as bridges connecting these two populations during the last glaciation (Speed 1981; Humphrey 1997; Graham 2003). Thus, we suggest that future studies on systematics and biogeography of Caribbean *Myotis* should focus on the definition of the taxonomic status of island populations from Grenada and Guadeloupe.

With the recognition of *M.larensis* as a full species, 28 species of Neotropical *Myotis* (sensu Stadelmann et al. 2007) are currently recognized: *M.albescens* (É. Geoffroy, 1806), *M.ruber* (É. Geoffroy, 1806), *M.nigricans* (Schinz, 1821), *M.levis* (I. Geoffroy, 1824), *M.chiloensis* (Waterhouse, 1840), *M.oxyotus* (Peters, 1866), *M.atacamensis* (Lataste, 1892), *M.nesopolus* Miller, 1900, *M.simus* Thomas, 1901, *M.dinellii* Thomas, 1902, *M.dominicensis* Miller, 1902, *M.caucensis* Allen, 1914, *M.keaysi* J.A. Allen, 1914, *M.riparius* Handley, 1960, *M.elegans* Hall, 1962, *M.larensis* LaVal, 1973, *M.martiniquensis* LaVal, 1973, *M.pilosatibialis* LaVal, 1973, *M.nyctor* LaVal & Schwartz, 1974, *M.diminutus* Moratelli & Wilson, 2011, *M.lavali*Moratelli et al., 2011, *M.izecksohni*Moratelli et al., 2011, *M.handleyi*Moratelli et al., 2013, *M.midastactus* Moratelli & Wilson, 2014, *M.clydejonesi*Moratelli et al., 2016, *M.attenboroughi*Moratelli et al., 2017, *M.bakeri* Moratelli et al., 2019, and *M.armiensis* Carrión-Bonilla & Cook, 2020. However, our results indicate that there are at least four haplogroups that might correspond to undescribed species. This scenario confirms the Neotropical region as a highly diverse region for *Myotis*.

## Appendix 1

List of specimens examined in the American Museum of Natural History (AMNH, New York, USA); Carnegie Museum of Natural History (CM, Pittsburgh, USA); Field Museum of Natural History (FMNH, Chicago, USA), Louisiana State University, Museum of Zoology (LSUMZ, Baton Rouge, USA); Museu de Zoologia da Universidade de São Paulo (MZUSP, São Paulo, Brazil); Museum of Texas Tech University (TTU, Lubbock, USA); Museum of Vertebrate Zoology, University of California (MVZ, Berkeley, USA); National Museum of Natural History, Smithsonian Institution (USNM, Washington, D.C., USA); Natural History Museum of Los Angeles County (LACM, Los Angeles, USA); Natural History Museum, University of Kansas (KU, Lawrence, USA); and Royal Ontario Museum (ROM, Toronto, Canada). Specimens marked with asterisks were included in the morphometric multivariate analysis.

*Myotisalbescens* (*N* = 10). Venezuela: Trujillo, Valera, Río Motatán (USNM 370933); Apure, Pto. Páez, Río Cinaruco (USNM 373913); Bolívar, Río Supamo, 50 km SE El Manteco (USNM 387693); Miranda, 7 km E Río Chico, Nr. Pto. Tuy (USNM 387700); Amazonas, Capibara, 106 km SW Esmeralda, Brazo Casiquiare (USNM 409392, 409395); Amazonas, San Juan, 163 km ESE Pto. Ayacucho, Río Manapiare (USNM 409403, 409408, 409410, 409411).

*Myotisattenboroughi* (*N* = 13). Trinidad and Tobago: Tobago Island, Charlottesville, 1 km N of Pirate’s Bay, Saint John Parish (USNM 540692 [paratype], 540693 [holotype]); Tobago Island, St. Mary Parish, Hillsborough Reservoir (USNM 538064, 538065, 538066, 538067, 538068, 538069, 540619, 540620, 540621, 540694, 540695 [paratypes]).

*Myotiscaucensis* (*N* = 22): Colombia: Valle del Cauca, Cauca river (AMNH 32787 [holotype]); Valle del Cauca, Candelaria, Ingenio Mayangüez (USNM 461858–461867). Peru: Cuzco, Madre de Dios, 15 km E Puerto Maldonado, Reserva Cuzco Amazónico (KU 144288–144291); Loreto, Yarinacocha (LSUMZ 12252, 12254–12258).

*Myotisclydejonesi* (*N* = 1): Suriname: Sipaliwini, Raleigh Falls (TTU 109227 [holotype]).

*Myotisdiminutus* (*N* = 2): Ecuador: Los Ríos, Santo Domingo, 47 Km S (By Road), Río Palenque Science Center (USNM 528569 [holotype]). Colombia: Nariño, La Guayacana (LACM 18761).

*Myotishandleyi* (*N* = 27). Venezuela: Araguá, Rancho Grande Biological Station, 13 km NW Maracay (USNM 517503, 562923, 562924, 562925, 562926–562933, 562934, 562935, 562936, 562937); Distrito Federal, Pico Ávila, 5 km NE Caracas, near Hotel Humboldt (USNM 370932 [holotype]); Distrito Federal, Pico Ávila, 5 km NE Caracas, near Hotel Humboldt (USNM 370891 [paratype]); Miranda, Curupao, 5 km NW Guarenas (USNM 387723); Monagas, 3 km NW Caripe, near San Agustín (USNM 409391, 409429–409431, 409433, 409435, 409437, 409438).

*Myotiskeaysi* (*N* = 45). Venezuela: Araguá, Rancho Grande Biological Station, 13 km NW Maracay (USNM 370893–370895, 370898–370902, 370911–370913, 370915– 370922, 370924, 370926, 370929); Araguá, Rancho Grande Biological Station, 13 km NW Maracay (USNM 370927, 370928, 370930, 370931); Araguá, Pico Guayamayo, 13 km NW Maracay (USNM 521564); Araguá, Rancho Grande, Portachuelo (USNM 562920, 563005, 563006); Araguá, Rancho Grande (USNM 562921); Bolívar, Gran Sabana (USNM 130625, 130626); Carabobo, Montalban, 4 km NW Montalban, La Copa (USNM 441741, 441742); Distrito Federal, Los Venados, 4 km NW Caracas (USNM 370889); Distrito Federal, Pico Ávila, 5 km NNE Caracas, near Hotel Humboldt (USNM 370890); Distrito Federal, junction Puerto Cruz Highwayand Colonia Tovar Highway, 0.5 km W (USNM 562984); Guárico, Hacienda El Vira, 10 km NE Altagracia (USNM 387707); Miranda, San Andrés, 16 km SE Caracas (USNM 373920); Miranda, Curupao, 5 km NW Guarenas (USNM 387714–387716, 387718); Monagas, Caripe (USNM 534265).

*Myotislarensis* (*N* = 16). Venezuela: Lara, Río Tucuyo (AMNH 130709* [holotype]); Falcón, Capatárida, 6 km SSW (USNM 441710*, 441711*, 441728*, 441735*, 441736*, 441737*, 441740); Zulia, Nr. Cojoro, 35 km NNE Paraguaipoa (USNM 441721*). Guárico (TTU 48162, 48163, 48164, 48168, 48169, 48170); Barinas (CM 78645).

*Myotisnesopolus* (*N* = 26). Curaçao: Punda (USNM 101849 [holotype]); Willemstad, Scharloo (USNM 102158); Westpunt, 2.8 km S, 4.5 km E of (CM 52432, 5433*). Bonaire, 8.5 km N, 2 km Wkralendijk (CM 52203, 52204, 52205, 52206, 52207, 52208, 52209, 52211, 52212*, 52213, 52214, 52215, 52216*, 52217*, 52218*, 52219*, 52220*, 52221, 52222*, 52223*, 52224, 52225).

Myotiscf.nigricans (*N* = 23). Suriname: Para, Zanderij (CM 63933, 69053, 77699). Venezuela: Carabobo, Urama, 10 Km NW Urama, El Central (USNM 140447, 373921–373924, 373926, 373929, 373932, 373933, 373935, 373936, 373942, 373943, 373946, 373947, 373948, 373949, 373950, 441741, 441742).

*Myotisoxyotus* (*N* = 9). Venezuela: Amazonas, Cerro Duida, Cano Culebra, 50 km NW Esmeralda (USNM 405799); Amazonas, Cerro Neblina, Camp VII (USNM 560809–560811); Bolívar, Km. 125, 85 km SE El Dorado (USNM387712); Bolívar, El Pauji, 21 km NE Icabaru, El Pauji (USNM441750); Distrito Federal, Alto Ño León, 33 km SW Caracas (USNM 409427); Mérida, La Mucuy, 4 km E Tabay (USNM373919, 387705).

*Myotispilosatibialis* (*N* = 11). Trinidad and Tobago: Trinidad Island, St. George (TTU 5441). Honduras: Francisco Morazán, 1 km W Talanga (LACM 36879 [holotype]). Guatemala: Chimaltenago, Chocoyos (FMNH 41653, 41839, 41840, 41841, 41843, 41844, 41845, 41846, 73365).

*Myotisriparius* (*N* = 33). Costa Rica: Puntarenas, 5.3 km S (byroad) San Vito (CM 92491); Limon, Fila La Maquina (LSUMZ 12928). French Guiana: Paracou, near Sinnamary (AMNH 266376, 268591). Guyana: Barima-Waini, North West District (USNM 568021); Potaro-Siparuni, Iwokrama Field Station, Iwokrama Forest (ROM 112049); Potaro-Siparuni, Iwokrama Reserve, Burro Burro River, 25 km WNW of Kurupukari (ROM 107278, 114620); Potaro-Siparuni, Mount Ayanganna, First Plateau Camp (ROM 114688, 114689); Upper Takutu-Upper Essequibo, Gunn’sStrip (ROM 106773). Nicaragua: Chontales (KU 11228). Panamá: Darién, Tacarcuna Village Camp, Río Pucro (USNM 310255 [holotype], 310254, 310256, 310257 [paratypes]); Darién, Rio Paya, Mouth (USNM 306798); Panamá, Cerro Azul (USNM 306795); Chiriquí (USNM 331916); Bocas del Toro, Isla Popa, 1 Km SE Deer Island Channel (USNM 464368). Trinidad and Tobago: Trinidad Island,St. George (TTU 5467). Venezuela: Amazonas,Boca Mavaca, 84 km SSE Esmeralda, 7 km up Río Mavaca (USNM 405803, 405804); Amazonas, Capibara, 106 km SW Esmeralda, Brazo Casiquiare (USNM 409457); Amazonas, ca 2 km SE Cerro Neblina Base Camp (USNM 560625); Amazonas, Tamatama, Río Orinoco (USNM 405806); Apure, Nulita, 29 km SW Santo Domingo, Selvas de San Camilo (USNM 416584, 441746, 441748); Araguá, Rancho Grande (USNM 562940); Barinas, 7 km NE Altamira (USNM 441743); Bolívar, Río Supamo, 50 km SE El Manteco (USNM 387721); Bolívar, San Ignacio de Yhuruani (USNM 448544).

*Myotissimus* (*N* = 56). Brazil: Amazonas, Borba (AMNH 91886–91892, 94224, 94225, 94227, 94230–94234); Amazonas, Itacoatiara (MZUSP 4372); Amazonas, Manaus (AMNH 79534, 91472–91478, 91500); Amazonas, Parintins (AMNH 92983, 93489–93497, 93922–93925); Amazonas, Rio Juruá (MZUSP 638, 1074).

## Appendix 2

**Table:** Specimens used in cytochrome-b analyses, including terminal taxa (focal and putative species of *Myotis*), GenBank accession numbers of sequences, voucher specimens, localities of origin, and source of information. The information presented for terminal taxonomic identifications results from re-identification of specimens (see Materials and methods), and does not necessarily match those identifications assigned by researchers that generated the corresponding sequence(s) available at GenBank. Abbreviations and acronyms for institutional collections are as follows: American Museum of Natural History, New York, USA (AMNH), Carnegie Museum of Natural History, Pittsburg, USA (CM), Field Museum of Natural History, Chicago, USA (FMNH), Museum of Natural History, University of Kansas, Lawrence, USA (KU), Natural History Museum of Los Angeles County, Los Angeles, USA (LACM), Louisiana State University, Museum of Zoology, Baton Rouge, USA (LSUMZ), Museum of Vertebrate Zoology, University of California, Berkeley, USA (MVZ), University of Nebraska State Museum, Lincoln, USA (UNSM), Muséum national d’Histoire naturelle, Paris, France (MNHN), Národní Muzeum, Prague, Czech (NMP), Museo de Zoología de la Pontificia Universidad Católica del Ecuador, Quito, Ecuador (QCAZ), Royal Ontario Museum, Toronto, Canada (ROM), Universidad Autónoma Metropolitana, Iztapalapa, Mexico (UAMI), Universidade Federal Rural do Rio de Janeiro, Seropédica, Brazil (ALP); and Smithsonian National Museum of Natural History, Washington, DC, USA (USNM). Localities are arranged alphabetically by species and major political unities.

Terminal	GenBank	Voucher	Locality	Source
*M.albescens*	JX130444	CM 63920	Nickerie, Suriname	Larsen et al. (2012a)
	JX130463	TTU 85088	Pastaza, Ecuador	Larsen et al. (2012a)
	JX130464	TTU 85089	Pastaza, Ecuador	Larsen et al. (2012a)
	JX130465	TTU 85094	Pastaza, Ecuador	Larsen et al. (2012a)
	JX130522	TTU 85091	Pastaza, Ecuador	Larsen et al. (2012a)
	JX130472	TTU 102363	El Oro, Ecuador	Larsen et al. (2012a)
	JX130500	TTU 102348	El Oro, Ecuador	Larsen et al. (2012a)
	JX130501	TTU 103744	Guayas, Ecuador	Larsen et al. (2012a)
	JX130445	TTU 46343	Huánuco, Peru	Larsen et al. (2012a)
	AF376839	FMNH 162543	Tarija, Bolivia	Ruedi and Mayer (2001)
	JX130503	TTU 99124	Boquerón, Paraguay	Larsen et al. (2012a)
	JX130502	TTU 99801	Ñeembucú, Paraguay	Larsen et al. (2012a)
	JX130504	TTU 99818	Ñeembucú, Paraguay	Larsen et al. (2012a)
*M.atacamensis*	AM261882	MVZ 168933	Olmos, Peru	Stadelmann et al. (2007)
*M.attenboroughi*	JN020573	UNSM ZM–29470	St. George Parish, Tobago	Larsen et al. (2012b)
	JN020574	UNSM ZM–29483	St. George Parish, Tobago	Larsen et al. (2012b)
*M.chiloensis*	AM261888	–	Santiago, Chile	Stadelmann et al. (2007)
*M.clydejonesi*	JX130520	TTU 109227	Sipaliwini, Suriname	Larsen et al. (2012a)
*M.dinellii*	JX130475	TTU 66489	Córdoba, Argentina	Larsen et al. (2012a)
*M.dominicensis*	AF376848	–	St. Joseph’s Parish, Dominica	Ruedi and Mayer (2001)
	JN020554	TTU 31519	St. Joseph’s Parish, Dominica	Larsen et al. (2012b)
	JN020555	TTU 31507	St. Joseph’s Parish, Dominica	Larsen et al. (2012b)
	JN020556	TTU 31508	St. Joseph’s Parish, Dominica	Larsen et al. (2012b)
*M.larensis*	JN020569	TTU 48161	Guárico, Venezuela	Larsen et al. (2012b)
	JX130529	TTU 48162	Guárico, Venezuela	Larsen et al. (2012a)
	JX130530	–	Guárico, Venezuela	Larsen et al. (2012a)
	JX130531	TTU 48163	Guárico, Venezuela	Larsen et al. (2012a)
	JX130532	TTU 48164	Guárico, Venezuela	Larsen et al. (2012a)
	JX130533	TTU 48168	Guárico, Venezuela	Larsen et al. (2012a)
	JX130535	CM 78645	Guárico, Venezuela	Larsen et al. (2012a)
	JX130543	TTU 48169	Guárico, Venezuela	Larsen et al. (2012a)
	JX130543	TTU 48169	Guárico, Venezuela	Larsen et al. (2012a)
*M.lavali*	AF376864	MVZ AD50	Paraíba, Brazil	Ruedi and Mayer (2001)
*M.levis*	AF376853	FMNH 141600	São Paulo, Brazil	Ruedi and Mayer (2001)
*M.martiniquensis*	AM262332	–	Martinique	Stadelmann et al. (2007)
	JN020558	MNHN:2005–896	Le Morne–Rouge, Martinique	Larsen et al. (2012b)
*M.martiniquensis*	JN020557	MNHN:2005–895	GranďRivière, Martinique	Larsen et al. (2012b)
	JN020559	–	GranďRivière, Martinique	Larsen et al. (2012b)
	JN020560	MNHN:2008–974	GranďRivière, Martinique	Larsen et al. (2012b)
	JN020561	–	GranďRivière, Martinique	Larsen et al. (2012b)
*M.nesopolus*	JN020575	–	Bonaire, Netherlands Antilles	Larsen et al. (2012b)
	JN020576	–	Bonaire, Netherlands Antilles	Larsen et al. (2012b)
	JN020577	–	Bonaire, Netherlands Antilles	Larsen et al. (2012b)
*M.nigricans*	JX130450	TTU 34952	La Paz, Bolivia	Larsen et al. (2012a)
	JX130528	TTU 34953	La Paz, Bolivia	Larsen et al. (2012a)
	JX130455	TTU 95992	San Pedro, Paraguay	Larsen et al. (2012a)
	JX130496	TTU 99743	Presidente Hayes, Paraguay	Larsen et al. (2012a)
	JX130498	TTU 99046	Alto Paraguai, Paraguay	Larsen et al. (2012a)
	JX130499	TTU 99802	Ñeembucú, Paraguay	Larsen et al. (2012a)
	JX130539	TTU 99516	Concepciόn, Paraguay	Larsen et al. (2012a)
	JX130540	TTU 99151	Boquerón, Paraguay	Larsen et al. (2012a)
*M.nyctor*	JN020562	CM 83427	St. David Parish, Grenada	Larsen et al. (2012b)
	JN020563	TTU 109225	St. Thomas Parish, Barbados	Larsen et al. (2012b)
	JN020564	TTU 109226	St. Thomas Parish, Barbados	Larsen et al. (2012b)
	JN020565	TTU 109229	St. Thomas Parish, Barbados	Larsen et al. (2012b)
	JN020566	TTU 109224	St. Thomas Parish, Barbados	Larsen et al. (2012b)
	JN020567	TTU 109230	St. Thomas Parish, Barbados	Larsen et al. (2012b)
*M.oxyotus*	AF376865	FMNH 129208	Lima, Peru	Ruedi and Mayer (2001)
*M.pilosatibialis*	JX130449	TTU 47514	Yucatán, Mexico	Larsen et al. (2012a)
	JX130525	–	Yucatán, Mexico	Larsen et al. (2012a)
	AF376852	–	Yucatán, Mexico	Ruedi and Mayer (2001)
	JX130489	CM 55764	Vera Cruz, Mexico	Larsen et al. (2012a)
*M.elegans*	JX130479	TTU 84380	Atlántida, Honduras	Larsen et al. (2012a)
	JX130480	TTU 84138	Atlántida, Honduras	Larsen et al. (2012a)
*M.riparius*	AM261891	–	La Selva, Costa Rica	Stadelmann et al. (2007)
	JX130474	CM 78659	Bolívar, Venezuela	Larsen et al. (2012a)
	JX130473	CM 68443	Para, Suriname	Larsen et al. (2012a)
	JX130469	TTU 85344	Esmeraldas, Ecuador	Larsen et al. (2012a)
	JX130515	TTU 85345	Esmeraldas, Ecuador	Larsen et al. (2012a)
	JX130572	TTU 102681	Esmeraldas, Ecuador	Larsen et al. (2012a)
	JX130492	TTU 102883	Esmeraldas, Ecuador	Larsen et al. (2012a)
	JX130513	TTU 84870	Pastaza, Equador	Larsen et al. (2012a)
	JX130506	TTU 85090	El Oro, Equador	Larsen et al. (2012a)
	JX130516	QCAZ 11380	Chimborazo, Equador	Larsen et al. (2012a)
	JX130436	–	Huánuco, Peru	Larsen et al. (2012a)
	JX130481	TTU 46348	Huánuco, Peru	Larsen et al. (2012a)
	AF376866	MVZ AD119*	Pernambuco, Brazil	Ruedi and Mayer (2001)
	AF376867	MVZ AD472*	São Paulo, Brazil	Ruedi and Mayer (2001)
	AM262336	–	São Paulo, Brazil	Stadelmann et al. (2007)
	JX130485	TTU 99645	Paraguari, Paraguay	Larsen et al. (2012a)
	JX130486	TTU 94912	Canindeyu, Paraguay	Larsen et al. (2012a)
*M.riparius*	JX130488	TTU 122454	Canindeyu, Paraguay	Larsen et al. (2012a)
	JX130491	TTU 99378	Canindeyu, Paraguay	Larsen et al. (2012a)
*M.velifer*	EF222340	TTU 48587	Texas, USA	Baird et al. (2008)
	EU680299	TTU 44818	Texas, USA	Baird et al. (2008)
	JX130468	TTU 109261	Texas, USA	Larsen et al. (2012a)
	AF376870	MVZ 146766	Sonora, Mexico	Ruedi and Mayer (2001)
	JX130478	TTU 44816	Tamaulipas, Mexico	Larsen et al. (2012a)
	JX130438	UAMI 15306	Michoacán, Mexico	Larsen et al. (2012a)
	JX130462	UAMI 15304	Michoacán, Mexico	Larsen et al. (2012a)
	JX130589	UAMI 15305	Michoacán, Mexico	Larsen et al. (2012a)
	JX130592	–	Michoacán, Mexico	Larsen et al. (2012a)
	JX130477	TTU 60983	Santa Ana, El Salvador	Larsen et al. (2012a)
*M.vivesi*	AJ504406	–	Gulf of California, Mexico	Stadelmann et al. (2004)
	AJ504407	–	Gulf of California, Mexico	Stadelmann et al. (2004)
*M.yumanensis*	AF376875	MVZ 15585	California, USA	Ruedi and Mayer (2001)
*M.* sp. 1	JX130523	TTU 103803	El Oro, Ecuador	Larsen et al. (2012a)
	JX130541	TTU 103751	El Oro, Ecuador	Larsen et al. (2012a)
	JX130546	TTU 102760	El Oro, Ecuador	Larsen et al. (2012a)
	JX130547	TTU 102765	El Oro, Ecuador	Larsen et al. (2012a)
	JX130548	TTU 102487	El Oro, Ecuador	Larsen et al. (2012a)
	JX130549	TTU 102489	El Oro, Ecuador	Larsen et al. (2012a)
	JX130550	TTU 102490	El Oro, Ecuador	Larsen et al. (2012a)
*M.* sp. 2	JX130452	TTU 46347	Huánuco, Peru	Larsen et al. (2012a)
	JX130537	TTU 46344	Huánuco, Peru	Larsen et al. (2012a)
	JX130538	TTU 46346	Huánuco, Peru	Larsen et al. (2012a)
*M.* sp. 3	JX130493	TTU 61228	Valle, Honduras	Larsen et al. (2012a)
*M.* sp. 4	JN020570	CM 63933	Nickerie, Suriname	Larsen et al. (2012b)
	JN020571	CM 69053	Para, Suriname	Larsen et al. (2012b)
	JN020572	CM 77699	Para, Suriname	Larsen et al. (2012b)
**Outgroups**				
*M.brandtii*	AF376844	–	Neuhaus, Germany	Ruedi and Mayer (2001)
	AM261886	NMP PB 916	North west, Russia	Stadelmann et al. (2007)
	AY665139	–	Moscow, Russia	Tsytsulina et al. (2012)
	AY665168	–	Znojmo, Czech Republic	Tsytsulina et al. (2012)
*M.gracilis*	AB106609	–	Hokkaido, Japan	Kawai et al. (2003)
	AB243025	–	Hokkaido, Japan	Kawai et al. (2006)
	AB243026	–	Hokkaido, Japan	Kawai et al. (2006)
	AB243027	–	Hokkaido, Japan	Kawai et al. (2006)
	AB243028	–	Hokkaido, Japan	Kawai et al. (2006)
	AB243029	–	Hokkaido, Japan	Kawai et al. (2006)
	AB243030	–	Hokkaido, Japan	Kawai et al. (2006)
